# Morphological and genetic changes induced by excess Zn in roots of *Medicago truncatula* A17 and a Zn accumulating mutant

**DOI:** 10.1186/1756-0500-5-657

**Published:** 2012-11-28

**Authors:** Ricky W Lewis, Guiliang Tang, David H McNear

**Affiliations:** 1 Rhizosphere Science Laboratory, Department of Plant and Soil Science, University of Kentucky, Lexington, KY 40546, USA; 2 Gene Suppression Laboratory, Department of Plant and Soil Science, University of Kentucky, Lexington, KY 40546, USA; 3 Department of Biological Sciences, Michigan Technological University, Houghton, MI 49931, USA; 4 Ag Science Bldg, North1100 Nicholasville Road, Lexington, KY 40546-0091, USA

**Keywords:** *Medicago truncatula*, Abiotic stress, MicroRNA (miRNA), Zn stress, Translocation factor, QRT-PCR, Legume, Root architecture

## Abstract

**Background:**

Nutrient fluxes associated with legume-rhizobia symbioses are poorly understood and little is known regarding the influence of abiotic stresses on development and maintenance of N-fixing nodules and root system architecture (RSA). We examined effects of Zn on nodule development and structure, root architecture, and expression of nodulation-related miRNAs in *Medicago truncatula* and the mutant, *raz* (requires additional Zn).

**Findings:**

Excess Zn increased root and shoot associated Zn in both genotypes, however, *raz* plants had lower root associated Zn than WT plants. Roots of *raz* plants exposed to excess Zn had less volume, surface area, and total length compared to WT plants. *Raz* plants had lower lateral root number than WT plants. Excess Zn was found to increase root diameter in both genotypes. The Mn Translocation Factor (T*f*_Mn_) increased in response to Zn in both genotypes; this was more pronounced in *raz* plants. T*f*_Zn_ was higher in *raz* plants and reduced in both genotypes in response to Zn. Nodulation was not influenced by Zn treatment or plant genotype. MicroRNA166 was upregulated under excess Zn in WT plants.

**Conclusions:**

Neither the *raz* mutation nor Zn treatment affected nodulation, however, *raz* plants had altered RSA compared with WT and responded differently to Zn, implying the mutation potentially modulates RSA responses to Zn but doesn’t play a direct role in nodulation. MicroRNA166 was significantly induced in WT plants by excess Zn, warranting further investigation into the potential role it plays in controlling RSA.

## Findings

### Background

*Medicago truncatula* has been established as a model legume species because it has many desirable attributes, including, a small diploid genome, short generation time [1], and some level of transformability and regenerability in some genotypes [2]. There are also a number of genetic resources available, such as, ESTs, a nearly complete sequence of gene rich regions of the genome, and genetic and physical maps [2]. Wild-type *M. truncatula* plants are not known to accumulate or hyperaccumulate metals, however, *M. truncatula raz* (for requires additional Zn) is an ethyl methanesulfonate (EMS) generated, Zn accumulating mutant first characterized in 2003 [3]. This mutant has been shown to accumulate greater than 10,000 μg Zn/ g d. wt. associated with root tissues and greater than 400 μg Zn/ g d. wt. associated with shoot tissues when grown in nutrient solutions containing 3 μM Zn and 2 μM Mn [3]. When grown in nutrients solutions defined by the authors as adequate for wild-type (WT) plants, *raz* exhibits a high level of leaf necrosis and subsequent leaf loss, which is very similar to WT plants grown in Zn deficient conditions [3]. Both leaf necrosis and leaf loss were partially ameliorated by providing the plants with 3 μM Zn and 0.2 μM Mn, however, *raz* produced less biomass than WT plants regardless of Zn treatment [3]. Segregation analysis of progeny from crosses of WT *M. truncatula* and third generation *raz* plants revealed that a single, recessive gene is likely responsible for the *raz* phenotype [3]. The mutation was localized to the upper arm of linkage group 7, as defined by Kulikova et al. [4] through genetic mapping studies of *raz* x *M. truncatula* A20 populations [3]. The phenotype observed in *raz* plants is thought to be generated by a functional Zn deficiency [3]. The *raz* phenotype, as characterized by Ellis et al. [3], was observed in non-nodulated plants, therefore, the phenotypic consequences of the mutation in nodulated plants have not yet been adequately explored. Zinc toxicity has been found to have numerous affects on plant growth. Studies in wheat and cucumber have revealed that high Zn decreases percent germination and inhibits root elongation as well as hypocotyl and coleoptile growth [5]. Elevated Zn has also been found to reduce root elongation rate in 4-weeks-old seedlings of *Picea abies*[6] and in rye grass [7]. At pZn activity = 5.25 and 5.0, Ibekwe et al. [8] found nodulation to be inhibited in alfalfa and Zn induced root damage was thought to be the cause. Additionally, zinc toxicity was found to cause deposition of lipids on the lumen surface of xylem vessel walls and deposition of phenolic compounds on the walls of secondary vessel cells [9]. High Zn may also inhibit cell division and elongation and increase root diameter [10,11].

A number of studies have recently emerged covering the response of microRNAs (miRNAs) to nutrient availability [12-23]; [24], but to date none have reported Zn responsive miRNAs. MicroRNAs are small non-coding RNA (21 nucleotides long) that are important players in regulation of almost all aspects of plant development, and the RNA interference (RNAi) related pathways. Through RNAi machinery, genes are posttranscriptionally regulated by miRNA resulting in cleavage of mRNA, or inhibition of mRNA translation due to the binding of the miRNA to the 3' UTR (untranslated region) [25]. RNAi pathways are important during biological development [25] and in adaptive plant responses to nutrient stress [26]. Studies have revealed the role of miRNA in sulfur and phosphate homeostasis, as discussed by Chiou [26]. One miRNA, miRNA 399, is thought to be a long distance signal in phosphate homeostasis in *Arabidopsis*[18]. It has also been shown that miRNAs regulate the expression of genes responsible for Cu/Zn superoxide dismutase under Cu limiting conditions in *Arabidopsis*[27].

In shoots of *A. thaliana,* miR166 and the close relative miR165 have been found to be involved in regulating the class III homeodomain-leucine Zipper (HD-ZIP III) family of transcription factors [28]. The dynamic, tissue specific regulation of these two miRNAs along with their associated targets are thought to be essential for multiple aspects of proper shoot development including, shoot apical meristem and floral development [29], vascular development in *Arabidsopsis* inflorescences [28], radial patterning [30,31], and initiation of floral and lateral shoot meristems [32]. Less is known about the function of these miRNAs in roots. Carlsbecker et al. [33], reported findings in support of a role for miR166b and miR165a in xylem cell identity and development in *Arabidopsis*. The authors propose induction of SHR (SHORT ROOT) in the metaxylem is followed by intercellular signaling which leads to the induction of SCR (SCARECROW) in the endodermis, where SHR and SCR induce miR165a/miR166b. The miRNAs are then thought to become mobile and migrate toward the metaxylem, regulating HD-ZIP III activity, leading to definition of cellular identity. Interestingly, Carlsbecker et al. [33] found miR166a to be undetectable in *Arabidopsis* roots and cite similar findings in other studies, but in *M. truncatula,* miR166a was found to be expressed in roots and nodules and to be involved in vascular patterning and nodule and lateral root formation [34]. Boualem et al. [34], found 2x35S:*MtMIR166a* overexpression resulted in reduced lateral root and nodule formation accompanied by, and possibly resulting from, disorganized vascular bundling. Overexpression of *MtMIR166a* also led to reduced transcript levels of three of the MtHB sub-class of HD-ZIP III genes, MtCNA1, MtCNA2, and MtHB8, however, the exact function of these genes remains unknown [34]. Expression of miR166 and the HD-ZIP III targets within the nodule are not mutually exclusive, with the miRNA and the MtHBs exemplifying co-regulation with respect to space and time. MicroRNA169 has been implicated in drought response in rice [23] and *Arabidopsis*[35], as well as in N and P limitation [36]. During nodulation in *M. truncatula,* miR169 is essential for proper nodule development by restricting *MtHAP2-1* transcripts primarily to the meristematic region of the nodule through posttranscriptional cleavage and overexpression of miR169 leads to the inhibition of nodule development presumably due to the lack of temporal-spatial control over MtHAP2-1 expression [37]. Pant et al. [36] found many miR169s to be repressed during N limitation in *Arabidopsis* and suggested that this may be a mechanism for detecting N deficiency and at least partially initiating nodulation through the mechanisms proposed by Combier et al. [37]. Rapid drops in miR169 transcript levels in phloem sap in response to N and P limitation also indicate the possibility of miR169 as a long-distance signal, whereby N and P deficiency are first detected in the shoots [36]; currently this hypothesis remains untested. As described above, miR166 and miR169 are thought to be involved in different aspects of nodulation. MicroRNA166 is likely involved in vascular bundling and it is thought that positional information derived from the stele is involved in lateral root and nodule development [38,39]. MiR166 might also be involved later in nodule development through tight regulation of MtHB expression [34]. The involvement of miR169 in nodulation is thought to be through maintenance of the meristematic region by restricting MtHAP2-1 expression primarily to this developmental region [37]. The objectives of this study were to examine root architectural responses and nodule developmental processes associated with Zn in wild type *Medicago truncatula* or a *raz* mutant. We examined nodule development and structure over a 28 day time course, recorded whole root system parameters, examined metal concentrations associated with shoot and root tissues, and quantified Zn responses of the nodulation-related miRNAs, miR166 and miR169, in nodulated WT and *raz* plants exposed to ideal and excess Zn. The root and nodule morphological data were gathered by using a combination of confocal microscopy and WinRHIZO image analysis software. MiRNA expression levels were quantified using qRT-PCR. In attempting to develop Zn fortified legume crops, it will be very beneficial to understand the influence of Zn on root architecture and nodule formation. Studying miRNA expression in *M. truncatula* will give us insights into gene regulation that is applicable to many N-fixing legumes, (i.e. Soybean*,* Chickpea, Lentil, Common Bean), which provide essential oils and proteins with little nitrogen input compared to non-leguminous crops. Exploration of the role of miRNA in symbiotic nodule development in *M. truncatula* has led to the identification of many genes and their associated miRNAs essential to nodulation [34,37,40].

### Materials and methods

#### Plant growth

*M. truncatula raz* and A17 seedlings were grown in a modified RainForest™ 236 aeroponic growth system (General Hydroponics, Sebastopol, CA, USA) (Additional file 1: Figure S1a). The Rainforest ™ system comes equipped with a vortex style pump, (Additional file 1: Figure S1b) which is ideal for hydroponic experiments requiring microbial inoculation as it minimizes microbial death associated with impeller type pumps that macerate the microbes or create too great a pressure differential. However, we found that the water droplet size released from the pump was too large for *M. truncatula* resulting in diminished lateral root formation, root growth and nodule number. To reduce the droplet size we attached a 125 μm polypropylene mesh around the circumference of the pump bracket (Additional file 1: Figure S1c) which reduced the spray to a fine mist. The smaller nutrient solution droplets promoted development of more lateral roots and generally enhance root growth, nodulation and plant health. Plant inserts accompanying the RainForest™ 236 unit were replaced with slimmer panels constructed from black acrylic through which 35, 5/16” holes were drilled every inch to accommodate plants (Additional file 1: Figure S1d). These panels can be easily lifted to access root tissue (Additional file 1: Figure S1e). The hydro units and all materials used for the preparation of nutrient solutions were acid washed in 10% HCl or 10% HCl-10% HNO_3,_ and further, the hydro units were surface sterilized and allowed to dry prior to sowing plants. WT and raz seeds were acid scarified and surface sterilized and then put on plates to germinate overnight in a dark drawer at room temperature. The next day plants of each genotype were sown into three black plant supports (Additional file 1: Figure S1d) by inserting their radical through the 5/16” hole. Prior to sowing the panels were covered with plastic wrap to support seedlings and provide a barrier to water loss or contamination to the reservoir. To maintain adequate humidity the system was outfitted with clear plastic domes and the plant foliage periodically sprayed with DDI water. Plants were grown at Room Temperature ± 3°C with 14 hours light (~5,382 lx) and 10 hours dark. Plantlets were grown in N replete conditions for five days, then N-starved for 5 days. On the sixth day after starving plants, solutions were inoculated with *Sinorhizobium meliloti*. Zn exposures were performed in modified 1/2x Lullien solutions (Lullien et al., 1987) at pH 6.5. “Ideal Zn” = 0.35 μM Zn (pZn activity ~9.85 with N and ~9.84 without N) and “High Zn” = 18 μM Zn (pZn activity ~7.55 with and without N) as calculated by GeoChem-EZ [41]. The nutrients and concentrations in Table 1 were entered into the software and free activities were estimated using the following criteria: fixed pH = 6.5, convergence criterion = 1e-4, number of iterations = 50, solids were allowed to precipitate, and ionic strength was estimated using a guess of 0.1 M/L (Table 2). Na_2_-EDTA concentrations were altered to augment Zn activities and maintain similar activities of other ions across treatments.

**Table 1:** **Nutrient solution modified from Lullien et al.**[47]

	**Modified Lullien Solution**			
**Compound**	**0.5x**	**0.5x High Zn**	**0.5x -N**	**0.5x High Zn -N**
**K**_**2**_**SO**_**4**_	0.26 mM	0.26 mM	0.26 mM	0.26 mM
**MgSO**_**4**_	0.125 mM	0.125 mM	0.125 mM	0.125 mM
**Na**_**2**_**-EDTA**	***25 μM***	***27 μM***	***25 μM***	***27 μM***
**H**_**3**_**BO**_**3**_	15 μM	15 μM	15 μM	15 μM
**MnSO**_**4**_	5 μM	5 μM	5 μM	5 μM
**ZnSO**_4_*7H_**2**_**O**	***0.35 μM***	***18 μM***	***0.35 μM***	***18 μM***
**CuSO**_**4**_	0.1 μM	0.1 μM	0.1 μM	0.1 μM
**Na**_**2**_**MoO**_**4**_	0.5 μM	0.5 μM	0.5 μM	0.5 μM
**CoCl**_**2**_	0.02 μM	0.02 μM	0.02 μM	0.02 μM
**CaCl**_**2**_	0.5 mM	0.5 mM	0.5 mM	0.5 mM
**NH**_**4**_**NO**_**3**_	2.5 mM	2.5 mM	--	--
**KH**_**2**_**PO**_**4**_	1.375 mM	1.375 mM	1.375 mM	1.375 mM
**K**_**2**_**HPO**_**4**_	1.375 mM	1.375 mM	1.375 mM	1.375 mM
**Fe(SO**_**4**_)*7H_**2**_**0**	25 μM	25 μM	25 μM	25 μM

Bold and italicized values indicate changes in concentration necessary for treatments, -- indicates nutrients were absent.

**Table 2:** **Results of GeoChem-EZ analysis of nutrients solutions in Table**1

	**Free Activity (−log)**			
**Ion**	**1/2x**	**1/2 High**	**1/2x -N**	**1/2x High -N**
Mg	4.176	4.177	4.16	4.16
Fe +2	5.902	5.818	5.894	5.822
Mn +2	6.189	5.801	6.178	5.79
Na	4.337	4.304	4.331	4.299
**Zn**	**9.847**	**7.552**	**9.838**	**7.551**
Cu +2	12.691	12.106	12.682	12.105
Co +2	11.09	10.506	11.081	10.505
Ca	3.563	3.563	3.546	3.545
K	2.378	2.378	2.372	2.372
**EDTA**	**14.984**	**15.571**	**14.968**	**15.547**
B(OH)4	7.522	7.522	7.521	7.521
Cl	3.044	3.044	3.038	3.038
SO4	3.585	3.568	3.565	3.547
MoO4	6.478	6.478	6.455	6.455
NH3	5.146	5.146	--	--
NO3	2.646	2.646	--	--
PO4	9.273	9.275	9.266	9.268

Activities are estimated -log free activity. Zn and EDTA are in bold because concentrations of these compounds were altered to achieve sufficient Zn and excess Zn.

#### RNA Isolation and qRT-PCR

Plants were harvested at two time points, 10 dpi, while nodules are developing, and at 15 dpi, when WT nodules should be mature [42]. Upon harvesting, plants were separated into roots and shoots, massed and flash frozen in liquid N_2_, with any damaged or dying tissue being removed. Samples were stored in −80°C until further processing. Total RNA containing microRNA was isolated from whole root tissues of 3 bioreps (5 plants each) using the mirVana™ miRNA Isolation Kit (Ambion, Austin, TX, USA) according to the manufacturer’s instructions. Quantity and quality of total RNA was assessed using a Cary 50 UV–vis spectrophotometer (Varian Australia Pty Ltd., USA). DNase treatment was performed on 10 μg total RNA in 50 μl reactions containing 2 μl DNase using TURBO™ DNase (Ambion, Austin, TX, USA). Reverse transcription was performed on 1 μg total RNA using the High Capacity cDNA Reverse Transcription Kit (Applied Biosystems, Foster City, CA, USA) according to manufacturer’s protocol. The same kit and RNA quantity was used for miRNA cDNA synthesis but random primers were replaced with 1 μM stem-loop primers specific to miR166 and miR169. The pulsed reverse transcription method described by Varkonyi-Gasic et al. [43] was used to generate cDNA for each miRNA in separate reactions. QRT-PCR was performed using 20 μl Power SYBR Green Master Mix reactions containing 2 μl of 1/10 diluted cDNA and the Applied Biosystems StepOnePlus 96-well plate system (Foster City, CA, USA) (See Additional file 2: Table S1 for primer concentrations). To amplify miR166 and miR169 along with the reference mRNA gene, actin-11, simultaneously it was necessary to modify the method established by Chen et al. [44] and refined by Varkonyi-Gasic et al. [43]. In short, the 1 sec extension step at 72°C was adjusted to 10 seconds which aided in achieving adequate primer efficiencies (Additional file 2: Table S1), particularly for actin-11. Primers were obtained from IDT (Integrated DNA Technologies, USA). All miRNA real-time primers and RT primers were designed according to methods developed by Chen et al. (2005). Primer sequences for actin-11 were used from Boualem et al. [34]. Fast PCR [45] was used to design and test (*in silico*) real-time primers. Sensitivity for primer detection was set to “3” as advised by the programmers. Amplification specificity was determined via melt-curve analysis and observing products on 4% agarose gel in TBE buffer. Mortars, pestles, and spatulas used in RNA extraction were washed and baked at 180°C overnight and RNase Zap (Ambion, Austin, TX, USA) was used to reduce the likelihood of RNase contamination. Real-time data were analyzed using GenEx Pro gene expression analysis software (version 5.2.7.44) and Systat and SAS statistical analysis software. The delta-delta Ct method was used to determine relative expression. The average relative expression of all treatments is set to zero, thus, values above zero indicate greater than average expression and values below zero indicate less than average expression. Standard error is the error of each biogroup (composed of 3 bioreps each). The Tukey’s post-hoc analysis was used to evaluate statistical differences between groups.

#### Confocal light microscopy

Nodule development and bacterial occupancy were monitored via confocal microscopy using SYTO13 (Molecular Probes, Inc., Eugene, OR, USA) as described by Haynes et al. [46]. Briefly*,* root sections containing nodules were harvested into a Petri dish containing 80 mM PIPES buffer at 7, 14, 21, and 28 days post inoculation (dpi). A 7x-45x trinocular stereo zoom microscope (AmScope, Chino, Ca, USA) was used to visualize the nodules while they were isolated from the root segments and bisected lengthwise using a double-edged razorblade. Bisected nodule halves were then transferred to 1.5 ml microcentrifuge tubes containing 80 mM PIPES buffer. Bisects were stained at room temperature for approximately 15–20 minutes in 1 ml of 80 mM PIPES buffer containing 3 μM SYTO 13 (Molecular Probes, Inc., Eugene, OR, USA). Nodules were then rinsed thrice in 80 mM PIPES buffer to remove excess dye. Stained nodule bisects were transferred with a minimal amount of PIPES solution to appropriate slides (Corning, Inc., Corning, NY, USA). Confocal imaging was performed with a Leica TCS SP5 inverted laser scanning confocal microscope (Leica Microsystems, Exton, Pa.) at 7 dpi and with a Leica SP1 inverted confocal microscope (Leica Microsystems, Exton, Pa.) for the remaining time points. In both cases, SYTO 13 excitation was achieved with an Argon helium laser (488 nM). Images were obtained in single layers (2-D) and multiple layers (3-D) depending on nodule orientation and the desired information. ImageJ (MacBiophotonics) was used to render single maximum-intensity projections, to scale the images, and to perform general analyses. Nucleic acid distribution (and hence bacterial occupancy) is depicted as green in the confocal images. Fluorescent intensities were adjusted to minimize maximum intensities, then contrast was normalized at +20%.

#### Total metal analysis

At 24 dpi, raz and WT plants grown in High and Ideal Zn were harvested and separated into roots and shoots. Tissue was massed and washed in 0.001 M CaCl_2_ to remove weakly sorbed cations and samples stored at −20°C for future analysis. Prior to ICP-MS analysis samples were freeze-dried for 3 days and massed into 15 ml metal free polypropylene centrifuge tubes (VWR Scientific). Samples were acid digested using an open vessel microwave digestion system (CEM MARS Express, Mathews, NC). Digestions were performed in two steps, first in 750 μl of trace metal grade HNO_3_ at 100°C for 10 minutes and then, after samples were cooled, 250 μl of H_2_0_2_ was added and the samples were heated again at 100°C for 10 minutes. Digestions were then brought to 15 ml using DDI H_2_0. Concentrations of Zn, Mn, and Mo were determined using an Agilent 7500 series ICP-MS (Santa Clara, Ca).

#### Root morphology

At 10 dpi roots of *M. truncatula* WT and *raz* grown in High and Ideal Zn were excised and examined to determine the influence of Zn on root morphology. For each genotype x treatment group, the roots of 5 plants were scanned and several root parameters, including total root length, average diameter, and total surface area, were recorded via WinRhizo Pro (Regent Instruments, Quebec, Canada). Lateral root and nodule counts were taken interactively using scanned images.

### Results

#### Both the raz mutation and Zn treatment have little effect on nodule phenotype and most differences observed in root morphology are genotype dependent

**Figure 1:**
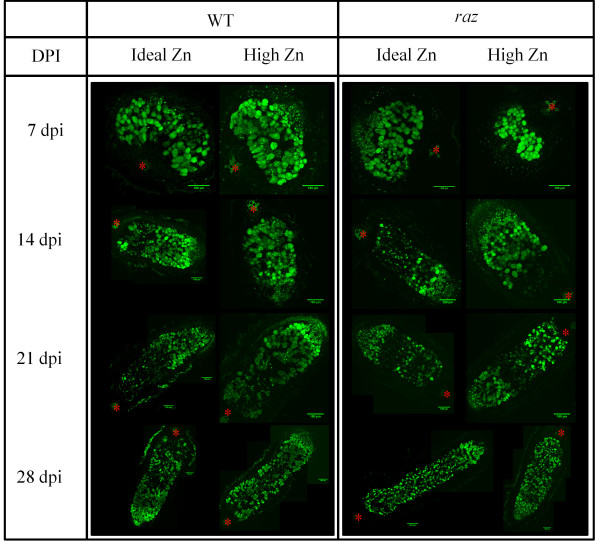
**Confocal images of *M. truncatula raz *and WT nodules at 7, 14, 21, and 28 dpi.** Images at 7 dpi were obtained with a Leica TCS SP5 Inverted Laser Scanning Confocal Microscope (Leica Microsystems, Exton, Pa.). Images at 14, 21, and 28 dpi were collected using a Leica SP1 Inverted Confocal Microscope (Leica Microsystems, Exton, Pa.). Green bars are 150 μm and red asterisks represent the stele of the root. SYTO 13, a green fluorescent nucleic acid specific stain was used to evaluate nodule development by tracking bacterial occupancy throughout the various transformations taking place during nodule formation. Some images are montages.

**Figure 2:**
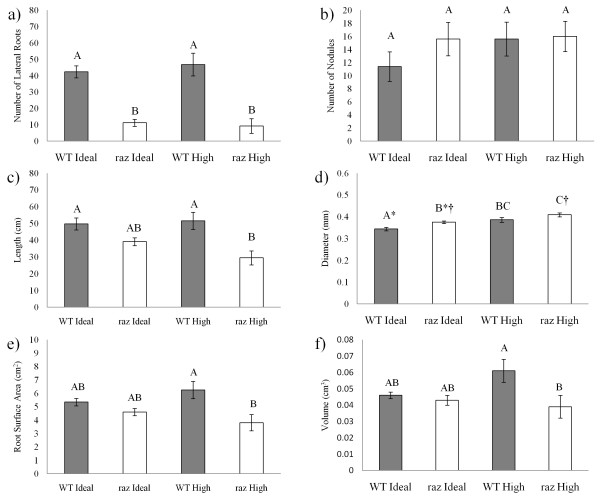
**Root parameters of *Medicago truncatula*.** Wild-type (grey columns) and *raz* (open columns) observed at 10 dpi using WinRhizo Pro. **a**) number of lateral roots, **b**) number of nodules, **c**) root length (cm), **d**) average root diameter (mm), **e**) root surface area (cm^2^), and **f**) root volume (cm^3^). Letter rankings indicate differences as determined by Tukey’s HSD at 95% confidence. Bars are standard error. * and † indicated trend evaluated at alpha = 0.1.

We examined nodules from WT and *raz* plants via confocal microscopy over a 28 day time course to determine the effects of Zn treatment on nodule development (Figure 1). We found no significant alteration in nodule development or structure in relation to Zn treatment or genotype in both *raz* and WT plants. *Raz* nodules in High and Ideal Zn treatments developed similarly to those of WT plants in the same treatment. This supports similar findings in *M. sativa,* where Zn was not found to alter nodule function, however, it is in conflict with results of Haynes et al. [46] which reported enlarged vacuoles in *raz* nodules. At 7 dpi, from nodule tip to stele, we observed the nodule meristem, prefixation zone and interzone (Figure 1). The quality of the confocal micrograph for the High Zn treated *raz* nodule at 7 dpi is poor, however, the micrographs for time points beyond this show no difference from those of the WT plants indicating that this was likely a sampling error and not a treatment effect. The N-fixation zone is expected to be underdeveloped at this stage. At 14 dpi we see the additional development of the N-fixation zone and the initiation of the senescence zone. From 14 to 28 dpi the senescence zone is expanding as the entire nodule elongates. Our studies did not consider nodule function, however, light microscope images of bisected WT and *raz* nodules appeared pink indicating the presence of leghemoglobin and the likelihood that nodules were able to fix N (unpublished data). Furthermore, Ibekwe et al. [8] found little difference in N_2_ fixation by nodules of *M. sativa* exposed to various levels of Zn as determined by acetylene reduction assays. Figure 2 shows the influence of Zn treatment and plant genotype on overall root system architecture at 10 dpi. *Raz* plants had significantly fewer lateral roots in Ideal and High Zn compared to WT (Figure 2a)*,* however, no genotype or treatment effect was observed with respect to the number of nodules (Figure 2b). For both WT and raz, we observed nodule numbers much lower than those recorded by Ibekwe et al. [8] in alfalfa (174 at pZn ~ 8) exposed to similar Zn activities; this may be a result of differences in the species or in experimental design. Root elongation was inhibited by excess Zn in *raz* compared to WT (Figure 2c.), implying the mutation potentially modulates root architectural responses to Zn in nodulated plants. As was also seen in *Quercus suber L*. seedlings by Disante et al. [10], exposure to High Zn resulted in increased root diameter in WT and *raz* plants compared to plants in Ideal Zn (Figure 2d). *Raz* plants in both treatments appear to have slightly greater root diameter compared to WT in respective treatments (Figure 2d). Root surface area and volume was greater in WT plants compared to *raz* plants in High Zn (Figure 2e,f).

#### Nodulation may dramatically influence concentrations of root associated Zn in *raz* plants

**Figure 3:**
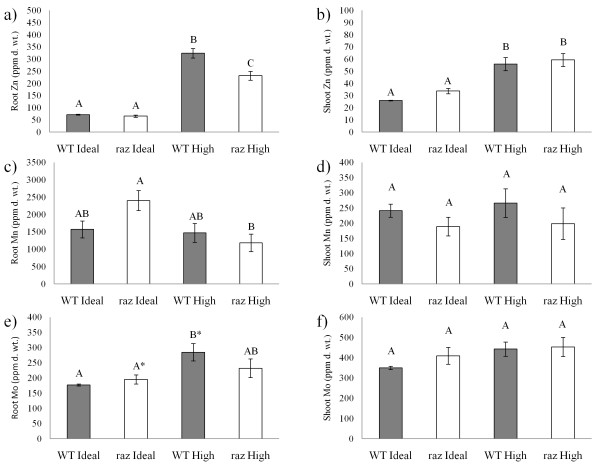
**Root and shoot tissue concentrations (ppm d. wt.) of Zn, Mn, and Mo at 24 dpi.***Medicago truncatula* wild-type (gray columns) and *raz* (open columns). **a**) is root Zn , **b**) is shoot Zn, **c**) is root Mn, **d**) is shoot Mn, **e**) is root Mo and **f**) is shoot Mo. Letter rankings indicate differences as determined by Tukey’s HSD at 95% confidence. Bars are standard error. * indicates trend evaluated at alpha = 0.1.

**Figure 4:**
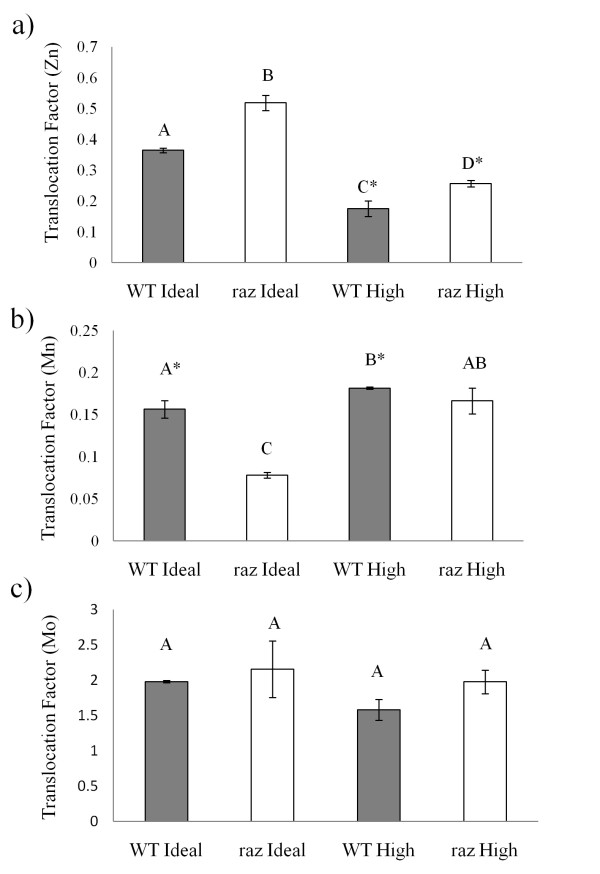
**Translocation Factor (T*f*).** Zn (**a**), Mn (**b**), and Mo (**c**) (*Tf* = [*Metal*_*shoot*_]/[*Metal*_*root*_]) at 24 dpi in *Medicago truncatula* wild-type (gray columns) and *raz* (open columns). Letter rankings indicate differences as determined by Tukey’s HSD at 95% confidence. * indicates alpha = 0.1 for this pairwise comparison. Bars are standard error.

Due to our inability to nodulate plants in the nutrient solutions used by Ellis et al. [3] (pZn = 5.7 (3 μM)) we switched to a modified 1/2x Lullien solution [47], which is widely used by the *M. truncatula* community, supplemented with 18 μM Zn (pZn = 7.55) which supplied significantly higher than normal Zn to the plant but didn’t inhibit nodule formation (Table 1). The “Ideal” treatment had 0.35 μM Zn (pZn ~9.85) which is close to pZn activities in the typical 1x Lullien solution (~9.9) (Table 1). Zn activities were much higher in all Zn treatments (save no Zn) in the studies of Ellis et al. [3] and Lopez-Millan et al. [48] than in a standard 1x Lullien. In the nutrient solutions used by Ellis et al. [3] and Lopez-Millan et al. [48] the lowest Zn concentration tested (save no Zn) was [Zn] = 0.2 μM, at which pZn activity is ~6.9; roughly 3 orders of magnitude greater Zn activity than the standard 1x and ½x Lullien solutions. Using these growth conditions (0.35 and 18 μM), we found greater root associated Zn in nodulated WT plants (>320 ppm d. wt.) compared to nodulated *raz* plants (>230 ppm d. wt.) in the High Zn treatment (Figure 3a). This finding is different than that found by Ellis et al. [3] and Lopez-Millan et al. [48] in non-nodulated plants, suggesting nodulation may affect Zn uptake and allocation in *raz* plants. While shoot associated Zn showed no difference between genotypes in either treatment, excess Zn resulted in a statistically significant increase in both genotypes compared to Ideal Zn (Figure 3b). Since the pZn activities in nutrient solutions used in our studies are different than those used by Ellis et al. [3] and Lopez-Millan et al. [48] it is difficult to compare the actual tissue associated concentrations, although our results for root and shoot Zn concentrations in WT plants are similar in magnitude to theirs. The High Zn treatment effectively reduced the Zn Translocation Factor (T*f*_(Zn)_ = [Zn_shoot_/[Zn_root_) in both genotypes (Figure 4a), which is expected because plants are known to tolerate metal concentrations beyond their physiological requirements by sequestering the metals either in or on the roots, reducing translocation to the shoots [11]. Interestingly, *raz* plants had greater translocation of Zn to the shoots under both Zn conditions compared to WT (Figure 4a). Ellis et al. [3] and Lopez-Millan et al. [48] found *raz* plants had greater root tissue associated Zn and Mn levels than WT plants under various Zn-Mn regimes and that *raz* plants appear to take up more Mn with increasing Zn concentrations, with the exception of [Zn] = 0. As such we monitored Mn concentrations in our nodulated treatments. Nodulated *raz* plants in the High Zn condition had lower root associated Mn levels (>1,180 ppm d. wt.) than in the Ideal Zn condition (~2,400 ppm d. wt.), while there was no difference in root Mn concentration between treatments in the WT plants (Figure 3c). Shoot associated Mn showed no statistical differences with respect to genotype or treatment (Figure 3d). In this study, r*az* plants in Ideal Zn had a lower T*f*_(Mn)_ compared to WT plants in Ideal Zn (Figure 4b). The *raz* plants also had a lower T*f*_(Mn)_ compared to both plant genotypes in High Zn (Figure 4b). WT plants appear to show only a slightly greater T*f*_(Mn)_ in High Zn compared to Ideal Zn (alpha = 0.1) (Figure 4b). From these observations, it appears that Mn translocation is affected by Zn much more dramatically in *raz* plants and that the High Zn condition results in a T*f*_(Mn)_ similar to that of WT in Ideal and High Zn. Molybdenum is an important cofactor in the nitrogenase enzyme complex which is essential to N fixation in legumes, as such, tissue associated Mo was measured to ensure the nutritional status of the plant was adequate to support N-fixation in raz and WT plants. Surprisingly, we found shoot and root molybdenum levels (Figure 3e,f) well above 5 ppm, the “maximum tolerable concentration” determined for beef cattle [49] even at the low Mo activities (pMo activity ~6.5) used in this study which were similar to those used by Ellis et al. [3] and Lopez-Millan et al. [48]. As noted by Gupta [50], there is little research on Mo toxicity because it rarely occurs in plants and no definite legume toxicity concentration could be found in the literature by the authors of this work, however, similar levels of Mo have been observed in alfalfa grown in mine tailings [51]. Plants used in this study did not exhibit any signs of Mo toxicity as defined in the close relative *M.sativa* (i.e. yellowing and eventual bronzing of the leaves) [50]. No trends were noticed in shoot associated Mo with regards to treatment or genotype and there were no genotype or treatment effects found with regards to T*f*_(Mo)_ (Figure 4c). However, WT plants grown under excess Zn showed significantly more Mo associated with roots (>280 ppm d. wt.) compared to WT (>170 ppm d. wt.) and *raz* (>195 ppm d. wt., alpha = 0.1) plants under Ideal Zn (Figure 3e,f). *Raz* plants showed no statistical increase in root associated Mo concentrations with respect to High Zn, which may indicate an association between the *raz* mutation and Mo uptake in response to Zn. Previous researchers found no relationship between Zn and Mo levels associated with plant tissues of *Trifolium pratense* L [52], so the relationship we observed may not be universal to all plants.

#### qRT-PCR analysis shows mature miR166 is upregulated in response to Zn

**Figure 5:**
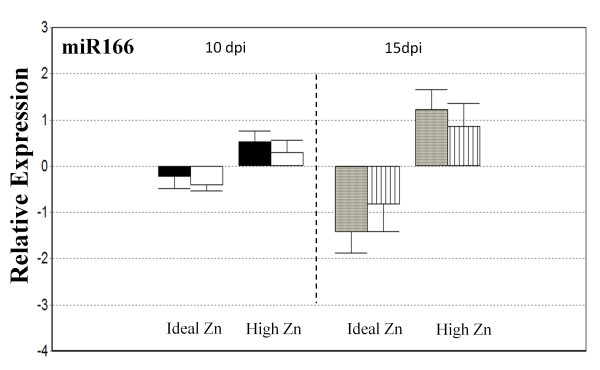
**Real-time RT-PCR analysis of miR166.***M. truncatula* WT (black and gray bars) and *raz* (white and striped bars). DPI is days post inoculation and bars are standard error. Expression is set relative to the average. Data is normalized to actin-11.

Expression of miR166 was observed in WT and raz plants under Ideal and elevated Zn conditions at 10 dpi and 15 dpi (Figure 5). Three-factor ANOVA analysis of miR166 relative expression data revealed a significant treatment effect (p-value ~ 0.0001) and treatment x day effects (p-value ~ 0.017). Due to treatment x day interactions 2-factor ANOVA analyses were performed at each time point. Elevated Zn led to statistically significant upregulation of miR166 at 15 dpi in the roots of nodulated *M. truncatula* WT and *raz* compared to nodulated WT roots in Ideal conditions (Figure 5). While *raz* plants at 15 dpi in High Zn do not demonstrate a statistically significant upregulation compared to *raz* plants in Ideal Zn at this time point, there is no difference between genotypes, indicating little difference in miR166 Zn responses between *raz* and WT. At 10 dpi miR166 is not statistically upregulated under elevated Zn conditions, nonetheless, given that miR166 is downregulated in control plants at the 15 dpi time point, our results indicate that elevated Zn induces miR166 and leads to maintenance of higher miR166 expression over the observed time frame (10-15dpi).

#### qRT-PCR analysis shows mature miR169 is downregulated at the 15 dpi time point

**Figure 6:**
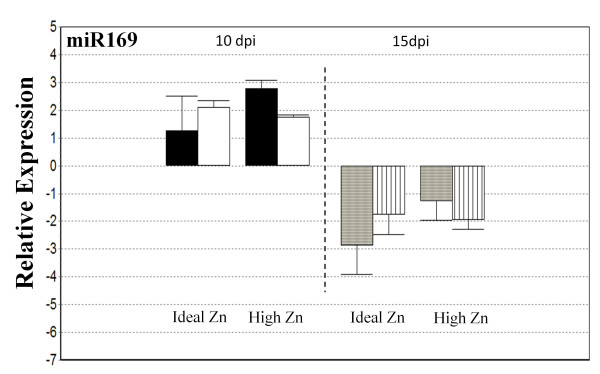
**Real-time RT-PCR analysis of miR169.***M. truncatula* WT (black and gray bars) and *raz* (white and striped bars). DPI is days post inoculation and bars are standard error. Expression is set relative to the average. Data is normalized to actin-11.

Expression levels of miR169 did not show any statistically significant trend in response to Zn in WT or *raz* plants at either time point (Figure 6). However, 3-way ANOVA analysis revealed a significant day effect. At 15 dpi, miR169 expression was downregulated with respect to 10 dpi at α = 0.05. Whole root tissue was used for both time points and our results imply that miR169 is downregulated at the 15 dpi time point in both plant genotypes under both Zn treatments. This result is complementary to the findings of Combier et al. [37] where the precursor, *MtMIR169a,* was downregulated at 14 dpi compared to 10 dpi in nodulated roots.

### Discussion

Exposure of WT and *raz* to excess zinc resulted in many differences in root system architecture between the two genotypes. Interestingly, differences in lateral root number were evident between genotypes but no difference was noticed in nodule number or development; implying that while the *raz* mutation does inhibit lateral root formation it does not inhibit the closely related process of nodulation. Ibekwe et al. [8] found high Zn activities delayed or inhibited nodulation in *Medicago sativa* inoculated with *S. meliloti*, but did not inhibit N-fixation in developed nodules. Conversely, Zn deficiency has been shown to disturb symbiotic N-fixation by altering other processes within the host plant, such as nutrient transport and assimilation [53]. Expression of a Krüppel-like zinc finger protein is integral to the formation of the N-fixation zone in *M. truncatula*[54]. In these ways, Zn plays a significant role in nodule development. In our study, the process of nodulation was unaffected by the Zn treatment in both genotypes.

Ellis et al. [3] found Zn tissue concentrations in non-nodulated *M. truncatula* WT and *raz* to be dependent upon Mn concentrations and vice versa, with *raz* exhibiting greater sensitivity to variations in concentration of either nutrient. For instance, at 3 μM Zn and 2 μM Mn, *raz* plants had root Zn levels >10,000 μg g^-1^ d. wt. compared with *raz* at 3 μM Zn and 0.2 μM Mn which had <4,000 μg g^-1^ d. wt. In contrast, WT plants showed little difference in root Zn levels in either of these treatments. Also, *raz* had greater root Mn levels at 3 μM Zn and 2 μM Mn (~3,000 μg g^-1^ d. wt.) and 0 μM Zn and 2 μM Mn (>4,000 μg g^-1^ d. wt.) compared with 1 μM Zn and 2 μM Mn (<2,000 μg g^-1^ d. wt. ). Under these and several other Zn:Mn ratios *raz* plants had greater root Zn and Mn concentrations than WT plants. Using the same nutrient solution with Zn and Mn concentrations (3 μM Zn and 0.2 μM Mn and pH 5.5) that Ellis et al. [3] showed to be ideal for *raz* in non-nodulated treatments, we made several unsuccessful attempts to nodulate WT and *raz* plants. To evaluate why the plants weren’t nodulating, we used GeoChem EZ to model the nutrient solutions used by Ellis et al. [3] and found the pZn activity to be around 5.7 at pH 5.5. Ibekwe et al. [8] showed that yellowing and necrosis in young leaves of *M. sativa* occurred at a pZn activity = 8, 5.25, and 5.0, with plant health improving after 10 days of treatment at pZn activity = 8. However, at pZn activity = 5.25 and 5.0 they found overall plant growth was stunted and no nodulation was achieved. Lack of nodulation was attributed to damaged root tissue. Ibekwe et al. [8] were examining the interaction of Cd/ Zn toxicity on *M. sativa* and *S. meliloti*, but the Zn studies were carried out at a Cd activity that was found to not influence the growth or nodulation of the plants or *rhizobia*. There is little research on the influence of metal concentrations on nodulation in *M. truncatula* so it is possible that the response to heavy metals may be slightly different than that of *M. sativa*. Regardless, we have shown some agreement with the influence of Zn on nodulation between the species.

Nodulation appears to alter the relationship between Zn and Mn uptake and allocation in *M. truncatula* compared to non-nodulated experiments shown in previous studies [3,48]. This relationship seems to be altered more dramatically in the *raz* mutant, where a greater proportion of Zn was allocated to the shoots in both High and Ideal Zn compared with WT and where a greater proportion of Mn remained associated with the roots in Ideal Zn (Figure 4xa, b). In our study, WT and *raz* plants had significantly higher levels of Zn in root and shoot tissues in the High Zn treatment compared to Ideal conditions (Figure 3a, b). Ellis et al. (2003) reported greater Zn concentration in roots verses shoots and elevated Zn conditions are known to influence metal distribution in the plant by inhibiting translocation of Zn to the shoots leading to Zn accumulation in the roots (Rout and Das 2003). Interestingly, WT plants had significantly more Zn associated with root tissues compared to *raz* under High Zn conditions. This finding is contrary to the results of Ellis et al. (2003) and Lopez-Millan et al. (2005) in non-nodulated plants, suggesting nodulation affects Zn uptake and distribution in *raz* plants. Since the pZn activities in nutrient solutions used in our studies are different than those used by Ellis et al. (2003) and Lopez-Millan et al. (2005) it is difficult to compare the actual tissue concentrations, though our results for root and shoot Zn concentrations in WT plants do fall roughly in the range that one may expect based on their results. Perhaps the lower levels of Zn in the roots is an artifact of the excess Zn being shuttled to the shoots (T*f*_Zn_ is higher in *raz*). It could also be related to the smaller size of *raz* roots given that surface area and volume are lower in this genotype. We also observed greater uptake of Mn by raz roots exposed to Ideal Zn versus High Zn (Figure 3c). Ellis et al. (2003) and Lopez-Millan et al. (2005) found raz plants had greater root tissue associated Zn and Mn levels than WT plants under various Zn-Mn regimes and that raz plants tend to take up more Mn with increasing Zn concentrations, with the exception of [Zn] = 0. These results again point to a possible influence of nodulation on the partitioning of metals within the plant and warrants further experimentation to deduce the mechanisms underlying this dramatic, possibly nodulation induced, change in Mn (and Zn) uptake by raz mutants.

There is much to learn concerning the nutrient demands generated by nodulation or the fluxes in nutrient uptake and allocation possibly associated with the formation and maintenance of nodules, as well as symbiosis as a whole. The potential nodulation-related changes we observed in Zn and Mn uptake in *M. truncatula* may be indicative of nutritional adjustments necessary to properly form and maintain the symbiosis. Previous studies in non-nodulated plants have found no correlation between MnSOD and total Mn associated with plant tissues of either genotype, however, a strong correlation was found between tissue associated Zn and ZnSOD [55]. The authors found ZnSOD levels were lower in raz plants exposed to high Zn compared with WT in the same treatment and compared with both genotypes in lower Zn, indicating that Zn is less available in raz roots past a certain threshold. Concentrations of root associated Mo were increased in response to Zn in WT but not raz. This may be indicative of differences in the plant genotypic responses to excess Zn. If raz plants are less sensitive to high levels of Zn, then the Zn tolerance mechanisms, such as the release of metal sequestering compounds into the rhizosphere, would be induced to a lesser extent compared with WT, thereby leading to lower levels of root associated Mo. However, since previous researchers have found no such association in other species and no work has been done regarding this type of interaction in Medicago species, this warrants further study.

As discussed earlier, excess Zn is known to affect several developmental parameters (i.e. increased root diameter, inhibition of root elongation, and inhibition of hypocotyl and coleoptile growth). Given that miR166 is thought to play roles in root cell identity [33], our findings, even though we are unable to determine which miR166 family member(s) is/are the source of the mature miRNA, may allude to part of the genetic mechanism by which Zn inhibits cell division. It is not uncommon for researchers to report increases in root diameter along with decreases in root elongation [56-59]. It is also thought that decreases in root elongation can potentially arise from decreases in cell division [59], understandably, given the intimate connection between the two processes [58]. Boualem et al. (2008) showed that *MtMIR166a* is involved in nodule development as well as root architecture in nodulated and non-nodulated *M. truncatula*. They showed a progressive trend in downregulation of *MtMIR166a* in nodules of *M. truncatula* moving from 1, 3, 8, and 21 dpi, with the 21 dpi point showing significant downregulation. Here, we are reporting two time points within this range and have found a similar trend in mature miR166 expression; downregulation from whole nodulated roots in Ideal Zn. Boualem et al. [34], also showed that overexpression of miR166 in *M. truncatula* via 2x35S promotion leads to a decrease in nodule number and lateral root formation accompanied by dramatic reordering of vascular bundling patterns. We did not investigate vascular bundling patterns, but we found no experimental effect on nodule number and no treatment effect on lateral root number (Figure 2a). We did note a genotypic difference in lateral root number, where WT plants generated significantly more lateral roots than *raz* plants (Figure 2a); the same phenomenon was observed in lateral root density (Additional file 3: Figure S2). Boualem et al. [34] examined the effects of overexpressing miR166 through strong promotion and our study was constructed to analyze the behavior of miR166 in response to Zn in non-transgenic plants. The upregulation we witnessed is likely inadequate to reproduce the drastic changes in phenotype observed by Boualem et al. [34] and is potentially indicative of other, more subtle, roles of miR166 in Zn response. The suggested role of miR166 directed regulation of HD-ZIP III transcription factors in vascular bundling and patterning insinuates Zn induction of miR166 is possibly related to changes in root vascular structure necessary to withstand, or induced by, the elevated Zn condition.

Investigation into the functions of miRNAs in plants is rapidly evolving, revealing their roles in a multitude of biological processes from basic development to abiotic stress response via long distance phloem transport. Here we have shown that miR166 is upregulated in response to chronic Zn exposure in nodulated *M. truncatula* WT and *raz*. The effect of Zn on miR166 is statistically significant at the 15 dpi sample point. Given that Zn exposure did not generate phenotypes similar to those with 2x35S:*MtMIR166a* overexpression [34], our results may imply a previously unidentified role for miR166 in Zn response. WT plants developed significantly more lateral roots than *raz* plants while miR166 expression was very similar across genotypes. Differences in lateral root formation were not associated with Zn treatment, yet upregulation of miR166 was. We found no relationship between Zn exposure and expression of miR169. We also found no difference in expression of miR169 between genotypes. Complimentary to other experiments monitoring expression of the precursor, *MtMIR169a*, we found mature miR169 to be downregulated at the 15 dpi point compared to the 10 dpi point [37]. Suggesting that expression of mature miR169 in whole nodule containing root tissues may mimic what has previously been observed in root nodules. However, we observed no difference in nodule formation in response to Zn or between genotypes and given miR169 expression is related to nodule development and function by limiting the expression of MtHAP2-1to the meristematic region [37] it is unlikely that examination of nodule tissue only would show any meaningful trends.

### Conclusions

In summary, nodule development appears to be unaffected by the *raz* mutation, suggesting the alteration in Zn partitioning induced by the mutation is of little consequence concerning this process. Excess Zn also appears to have little influence on nodule development or number in *M. truncatula*, as has been observed in *M. sativa.* The major root architectural Zn responses observed in this study were amongst genotypes, indicating the *raz* mutation modifies root development in relation to WT plants and that this modification alters phenotypic responses to Zn. We have provided evidence that nodulation may alter the relationship between Zn and Mn concentrations associated with root and shoot tissues and possibly hinder the Zn accumulating abilities previously characterized in *raz.* MiR166 is induced in roots of *M. truncatula* WT and *raz* in response to Zn and this appears to be maintained through time under chronic Zn exposure. Given the proposed mechanism by which miR166 is thought to regulate, at least partially, root cell identity; our results may imply some part of the mechanism by which excess Zn inhibits cell division. Further studies should investigate the role that miR166 plays in Zn responses to determine the precise function of this miRNA in chronic Zn exposure. The findings we report here provide insight into how legume roots respond to Zn and add to previous research which also found moderately high Zn levels to have little effect on nodule development, provided Zn concentrations were beneath the threshold where nodulation is drastically inhibited. Future research should focus on the nutritional demands that nodule development places upon legumes and how this may alter nutrient fluxes within the plant. Investigation into the roles of miRNA in Zn stress may also prove fruitful in attempts to further understand genetic mechanisms governing Zn induced phenotypic responses.

## Abbreviations

raz: Requires additional zinc; WT: Wild type; RSA: Root system architecture; miRNA: microRNA.

## Competing interests

There are no competing interests associated with this work.

## Authors’ contributions

RL is the primary author of the manuscript and conducted all experiments appearing in it, with the exception of metal concentration analysis via ICP-MS. GT aided in the conception of the study, development of the RNA methodology, and interpretation of the data. DM aided in the conception of the study, experimental design, interpretation of the data, and contributed greatly to revision of the manuscript. All authors read and approved the final manuscript.

## Supplementary Material

Additional file 1 **Figure S1.** Images of the modified General Hyrdoponics Rainforest 236 aeroponic system. Ranforest 236 system as purchased, b) bacteria friendly vortex pump used to circulate and aerate the nutrient solution (ruler is 16”), c) addition of 125 micron polypropylene mesh connected by Velcro™ to the top cover of the Rainforest system to reduced the size of the nutrient solution to a fine mist, d) top loading acrylic panel insert with 5/16” holes drilled every inch to accommodate ~35 plantlets, and e) an operating Rainforest^TM^ 236 with single grow panel lifted to reveal roots, nutrient solution and mist screen. Click here for file

Additional file 2 **Table S1.** Forward (FWD) and reverse (REV) primer concentrations and efficiencies used in qRT-PCR. Primer concentrations found to provide the most efficient amplification and the calculated efficiencies. Click here for file

Additional file 3 **Figure S2.** Lateral root density (# of lateral roots/cm) of ***Medicago truncatula.*** Wild-type (grey columns) and *raz* (open columns) observed at 10 dpi using WinRhizo Pro. Letter rankings indicate differences as determined by Tukey’s HSD at 95% confidence. Bars are standard error. Ideal Zn = 0.35 μM (pZn activity ~9.85 with N and ~9.84 without N) and High Zn = 18 μM (pZn activity ~7.55 with and without N as calculated by GeoChem-EZ). Click here for file

## References

[B1] Cook DR Medicago truncatula – a model in the making!: Commentary Curr Opin Plant Biol 1999 2 4 301 304 10.1016/S1369-5266(99)80053-3 10459004

[B2] Rose RJ Medicago truncatula as a model for understanding plant interactions with other organisms, plant development and stress biology: past, present and future Funct Plant Biol 2008 35 4 253 264 10.1071/FP07297 32688781

[B3] Ellis DR Lopez-Millan AF Grusak MA Metal physiology and accumulation in a Medicago truncatula mutant exhibiting an elevated requirement for zinc New Phytol 2003 158 1 207 218 10.1046/j.1469-8137.2003.00706.x

[B4] Kulikova O Gualtieri G Geurts R Kim DJ Cook D Huguet T de Jong JH Fransz PF Bisseling T Integration of the FISH pachytene and genetic maps of Medicago truncatula Plant J 2001 27 1 49 58 10.1046/j.1365-313x.2001.01057.x 11489182

[B5] Munzuroglu O Geckil H Effects of Metals on Seed Germination, Root Elongation, and Coleoptile and Hypocotyl Growth in Triticum aestivum and Cucumis sativus Arch Environ Contam Toxicol 2002 43 2 203 213 10.1007/s00244-002-1116-4 12115046

[B6] Godbold DL Hüttermann A Effect of zinc, cadmium and mercury on root elongation of Picea abies (Karst.) seedlings, and the significance of these metals to forest die-back Environ Pollut Ecol Biol 1985 38 4 375 381 10.1016/0143-1471(85)90108-4

[B7] Wong MH Bradshaw AD A Comparison of the Toxicity of Heavy Metals, Using Root Elongation of Rye Grass, Lolium Perenne New Phytol 1982 91 2 255 261 10.1111/j.1469-8137.1982.tb03310.x

[B8] Ibekwe AM Angle JS Chaney RL VanBerkum P Zinc and cadmium toxicity to alfalfa and its microsymbiont J Environ Qual 1996 25 5) 1032 1040

[B9] Robb J Busch L Rauser WE Zinc Toxicity and Xylem Vessel Wall Alterations in White Beans Ann Bot 1980 46 1 43 50

[B10] Disante KB Fuentes D Cortina J Response to drought of Zn-stressed Quercus suber L. seedlings Environ Exp Bot 2011 70 2–3 96 103

[B11] Rout GR Das P Effect of metal toxicity on plant growth and metabolism: I. Zinc Agronomie 2003 23 1 3 11 10.1051/agro:2002073

[B12] Allen E Xie ZX Gustafson AM Carrington JC microRNA-directed phasing during trans-acting siRNA biogenesis in plants Cell 2005 121 2 207 221 10.1016/j.cell.2005.04.004 15851028

[B13] Bari R Pant BD Stitt M Scheible WR PHO2, microRNA399, and PHR1 define a phosphate-signaling pathway in plants Plant Physiol 2006 141 3 988 999 10.1104/pp.106.079707 16679424 PMC1489890

[B14] Jones-Rhoades MW Bartel DP Computational identification of plant microRNAs and their targets, including a stress-induced miRNA Mol Cell 2004 14 6 787 799 10.1016/j.molcel.2004.05.027 15200956

[B15] Liu PP Montgomery TA Fahlgren N Kasschau KD Nonogaki H Carrington JC Repression of AUXIN RESPONSE FACTOR10 by microRNA160 is critical for seed germination and post-germination stages Plant J 2007 52 1 133 146 10.1111/j.1365-313X.2007.03218.x 17672844

[B16] Lu SF Sun YH Shi R Clark C Li LG Chiang VL Novel and mechanical stress-responsive microRNAs in Populus trichocarpa that are absent from Arabidopsis Plant Cell 2005 17 8 2186 2203 10.1105/tpc.105.033456 15994906 PMC1182482

[B17] Navarro L Dunoyer P Jay F Arnold B Dharmasiri N Estelle M Voinnet O Jones JDG A plant miRNA contributes to antibacterial resistance by repressing auxin signaling Science 2006 312 5772 436 439 10.1126/science.1126088 16627744

[B18] Pant BD Buhtz A Kehr J Scheible WR MicroRNA399 is a long-distance signal for the regulation of plant phosphate homeostasis Plant J 2008 53 5 731 738 10.1111/j.1365-313X.2007.03363.x 17988220 PMC2268993

[B19] Reyes JL Chua NH ABA induction of miR159 controls transcript levels of two MYB factors during Arabidopsis seed germination Plant J 2007 49 4 592 606 10.1111/j.1365-313X.2006.02980.x 17217461

[B20] Sunkar R Kapoor A Zhu JK Posttranscriptional induction of two Cu/Zn superoxide dismutase genes in Arabidopsis is mediated by downregulation of miR398 and important for oxidative stress tolerance Plant Cell 2006 18 8 2051 2065 10.1105/tpc.106.041673 16861386 PMC1533975

[B21] Sunkar R Zhu JK Novel and stress-regulated microRNAs and other small RNAs from Arabidopsis Plant Cell 2004 16 8 2001 2019 10.1105/tpc.104.022830 15258262 PMC519194

[B22] Zhang Z Wei L Zou X Tao Y Liu Z Zheng Y Submergence-responsive MicroRNAs are Potentially Involved in the Regulation of Morphological and Metabolic Adaptations in Maize Root Cells Ann Bot 2008 102 4 509 519 10.1093/aob/mcn129 18669574 PMC2701776

[B23] Zhao BT Liang RQ Ge LF Li W Xiao HS Lin HX Ruan KC Jin YX Identification of drought-induced microRNAs in rice Biochem Bioph Res Co 2007 354 2 585 590 10.1016/j.bbrc.2007.01.022 17254555

[B24] Lewis RW Mendu V McNear DH Tang G Jain SM, Brar DS Roles of MicroRNAs in Plant Abiotic Stress Molecular Techniques in Crop Improvement 2009 Netherlands: Springer 357 372

[B25] He L Hannon GJ Micrornas: Small RNAs with a big role in gene regulation Nat Rev Genet 2004 5 7 522 531 10.1038/nrg1379 15211354

[B26] Chiou TJ The role of microRNAs in sensing nutrient stress Plant Cell Environ 2007 30 3 323 332 10.1111/j.1365-3040.2007.01643.x 17263777

[B27] Yamasaki H Abdel-Ghany SE Cohu CM Kobayashi Y Shikanai T Pilon M Regulation of copper homeostasis by micro-RNA in Arabidopsis J Biol Chem 2007 282 22 16369 16378 10.1074/jbc.M700138200 17405879

[B28] Kim J Jung JH Reyes JL Kim YS Kim SY Chung KS Kim JA Lee M Lee Y Narry Kim V microRNA-directed cleavage of ATHB15 mRNA regulates vascular development in Arabidopsis inflorescence stems Plant J 2005 42 1 84 94 10.1111/j.1365-313X.2005.02354.x 15773855 PMC1382282

[B29] Jung J-H Park C-M MIR166/165 genes exhibit dynamic expression patterns in regulating shoot apical meristem and floral development in Arabidopsis Planta 2007 225 6 1327 1338 10.1007/s00425-006-0439-1 17109148

[B30] McConnell JR Emery J Eshed Y Bao N Bowman J Barton MK Role of PHABULOSA and PHAVOLUTA in determining radial patterning in shoots Nature 2001 411 6838 709 713 10.1038/35079635 11395776

[B31] Emery JF Floyd SK Alvarez J Eshed Y Hawker NP Izhaki A Baum SF Bowman JL Radial Patterning of Arabidopsis Shoots by Class III HD-ZIP and KANADI Genes Current biology: CB 2003 13 20 1768 1774 10.1016/j.cub.2003.09.035 14561401

[B32] Otsuga D DeGuzman B Prigge MJ Drews GN Clark SE REVOLUTA regulates meristem initiation at lateral positions Plant J 2001 25 2 223 236 10.1046/j.1365-313x.2001.00959.x 11169198

[B33] Carlsbecker A Lee JY Roberts CJ Dettmer J Lehesranta S Zhou J Lindgren O Moreno-Risueno MA Vaten A Thitamadee S Cell signalling by microRNA165/6 directs gene dose-dependent root cell fate Nature 2010 465 7296 316 321 10.1038/nature08977 20410882 PMC2967782

[B34] Boualem A Laporte P Jovanovic M Laffont C Plet J Combier JP Niebel A Crespi M Frugier F MicroRNA166 controls root and nodule development in Medicago truncatula Plant J 2008 54 5 876 887 10.1111/j.1365-313X.2008.03448.x 18298674

[B35] Li W-X Oono Y Zhu J He X-J Wu J-M Iida K Lu X-Y Cui X Jin H Zhu J-K The Arabidopsis NFYA5 Transcription Factor Is Regulated Transcriptionally and Posttranscriptionally to Promote Drought Resistance Plant Cell 2008 20 8 2238 2251 10.1105/tpc.108.059444 18682547 PMC2553615

[B36] Pant BD Musialak-Lange M Nuc P May P Buhtz A Kehr J Walther D Scheible W-R Identification of Nutrient-Responsive Arabidopsis and Rapeseed MicroRNAs by Comprehensive Real-Time Polymerase Chain Reaction Profiling and Small RNA Sequencing Plant Physiol 2009 150 3 1541 1555 10.1104/pp.109.139139 19465578 PMC2705054

[B37] Combier JP Frugier F de Billy F Boualem A El-Yahyaoui F Moreau S Vernie T Ott T Gamas P Crespi M MtHAP2-1 is a key transcriptional regulator of symbiotic nodule development regulated by microRNA169 in Medicago truncatula Gene Dev 2006 20 22 3084 3088 10.1101/gad.402806 17114582 PMC1635144

[B38] Beveridge CA Mathesius U Rose RJ Gresshoff PM Common regulatory themes in meristem development and whole-plant homeostasis Curr Opin Plant Biol 2007 10 1 44 51 10.1016/j.pbi.2006.11.011 17157052

[B39] Complainville A Brocard L Roberts I Dax E Sever N Sauer N Kondorosi A Wolf S Oparka K Crespi M Nodule Initiation Involves the Creation of a New Symplasmic Field in Specific Root Cells of Medicago Species Plant Cell 2003 15 12 2778 2791 10.1105/tpc.017020 14615602 PMC282798

[B40] Simon SA Meyers BC Sherrier DJ MicroRNAs in the Rhizobia Legume Symbiosis Plant Physiol 2009 151 3 1002 1008 10.1104/pp.109.144345 19789286 PMC2773061

[B41] Shaff JE Schultz BA Craft EJ Clark RT Kochian LV GEOCHEM-EZ: a chemical speciation program with greater power and flexibility Plant Soil 2010 330 1–2 207 214

[B42] Barker DGT Pfaff D Moreau E Groves S Ruffel M Lepetit S Whiteh F Maillet RM Nair Journet E Mathesius UJE, Sumner LW Growing M. truncatula: choice of substrates and growth conditions The Medicago truncatula handbook 2006 http://www.noble.org/medicago-handbook/

[B43] Varkonyi-Gasic E Wu RM Wood M Walton EF Hellens RP Protocol: a highly sensitive RT-PCR method for detection and quantification of microRNAs Plant Methods 2007 3 12 10.1186/1746-4811-1183-1112 PMC2225395 17931426

[B44] Chen CF Ridzon DA Broomer AJ Zhou ZH Lee DH Nguyen JT Barbisin M Xu NL Mahuvakar VR Andersen MR Real-time quantification of microRNAs by stem-loop RT-PCR Nucleic Acids Res 2005 33 20 e179 110.1093/nar/gni1178 16314309 10.1093/nar/gni178 PMC1292995

[B45] Kalendar R Lee D Schulman AH FastPCR Software for PCR Primer and Probe Desgin and Repeat Search Genes, Genomes and Genomics 2009 3 1 http://www.biocenter.helsinki.fi/bi/Programs/fastpcr.htm

[B46] Haynes JG Czymmek KJ Carlson CA Veereshlingam H Dickstein R Sherrier DJ Rapid analysis of legume root nodule development using confocal microscopy New Phytol 2004 163 3 661 668 10.1111/j.1469-8137.2004.01138.x 33873748

[B47] Lullien V Barker DG Delajudie P Huguet T Plant Gene-Expression in Effective and Ineffective Root-Nodules of Alfalfa (Medicago-Sativa) Plant Mol Biol 1987 9 5 469 478 10.1007/BF00015878 24277133

[B48] Lopez-Millan AF Ellis DR Grusak MA Effect of zinc and manganese supply on the activities of superoxide dismutase and carbonic anhydrase in Medicago truncatula wild type and raz mutant plants Plant Sci 2005 168 4 1015 1022 10.1016/j.plantsci.2004.11.018

[B49] NRC NRC Nutrient Requirements of Beef Cattle 1996 Washington, DC: National Academy Press

[B50] Gupta UC Deficient, Sufficient and Toxic Concentrations of Molybdenum in Crops 1997 New York, NY, USA: Cambridge University Press

[B51] Majak W Steinke D McGillivray J Lysyk T Clinical signs in cattle grazing high molybdenum forage J Range Manage 2004 57 3 269 274

[B52] McBride MB Richards BK Steenhuis T Spiers G Molybdenum Uptake by Forage Crops Grown on Sewage Sludge-Amended Soils in the Field and Greenhouse J Environ Qual 2000 29 3 848 854

[B53] Ohara GW Boonkerd N Dilworth MJ Mineral Constraints to Nitrogen-Fixation Plant Soil 1988 108 1 93 110 10.1007/BF02370104

[B54] Frugier F Poirier S Satiat-Jeunemaitre B Kondorosi A Crespi M A Kruppel-like zinc finger protein is involved in nitrogen-fixing root nodule organogenesis Gene Dev 2000 14 4 475 482 10691739 PMC316383

[B55] López-Millán AF Ellis DR Grusak MA Effect of zinc and manganese supply on the activities of superoxide dismutase and carbonic anhydrase in Medicago truncatula wild type and raz mutant plants Plant Sci 2005 168 4 1015 1022 10.1016/j.plantsci.2004.11.018

[B56] Vaughan D Ord B Influence of phenolic acids on morphological changes in roots of Pisum sativum J Sci Food Agric 1990 52 3 289 299 10.1002/jsfa.2740520302

[B57] Römheld V Marschner H Iron deficiency stress induced morphological and physiological changes in root tips of sunflower Physiol Plant 1981 53 3 354 360 10.1111/j.1399-3054.1981.tb04512.x

[B58] Inada S Tominaga M Shimmen T Regulation of Root Growth by Gibberellin in Lemna minor Plant Cell Physiol 2000 41 6 657 665 10.1093/pcp/41.6.657 10945334

[B59] Baskin TI Wilson JE Inhibitors of Protein Kinases and Phosphatases Alter Root Morphology and Disorganize Cortical Microtubules Plant Physiol 1997 113 2 493 502 10.1104/pp.113.2.493 9046596 PMC158165

