# Supplementary figures and images for: Morphological and genetic changes induced by excess Zn in roots of Medicago truncatula A17 and a Zn accumulating mutant
Source: BMC Res Notes. 2012 Nov 28;5:657. doi: 10.1186/1756-0500-5-657 (PMC3599297; doi:10.1186/1756-0500-5-657)

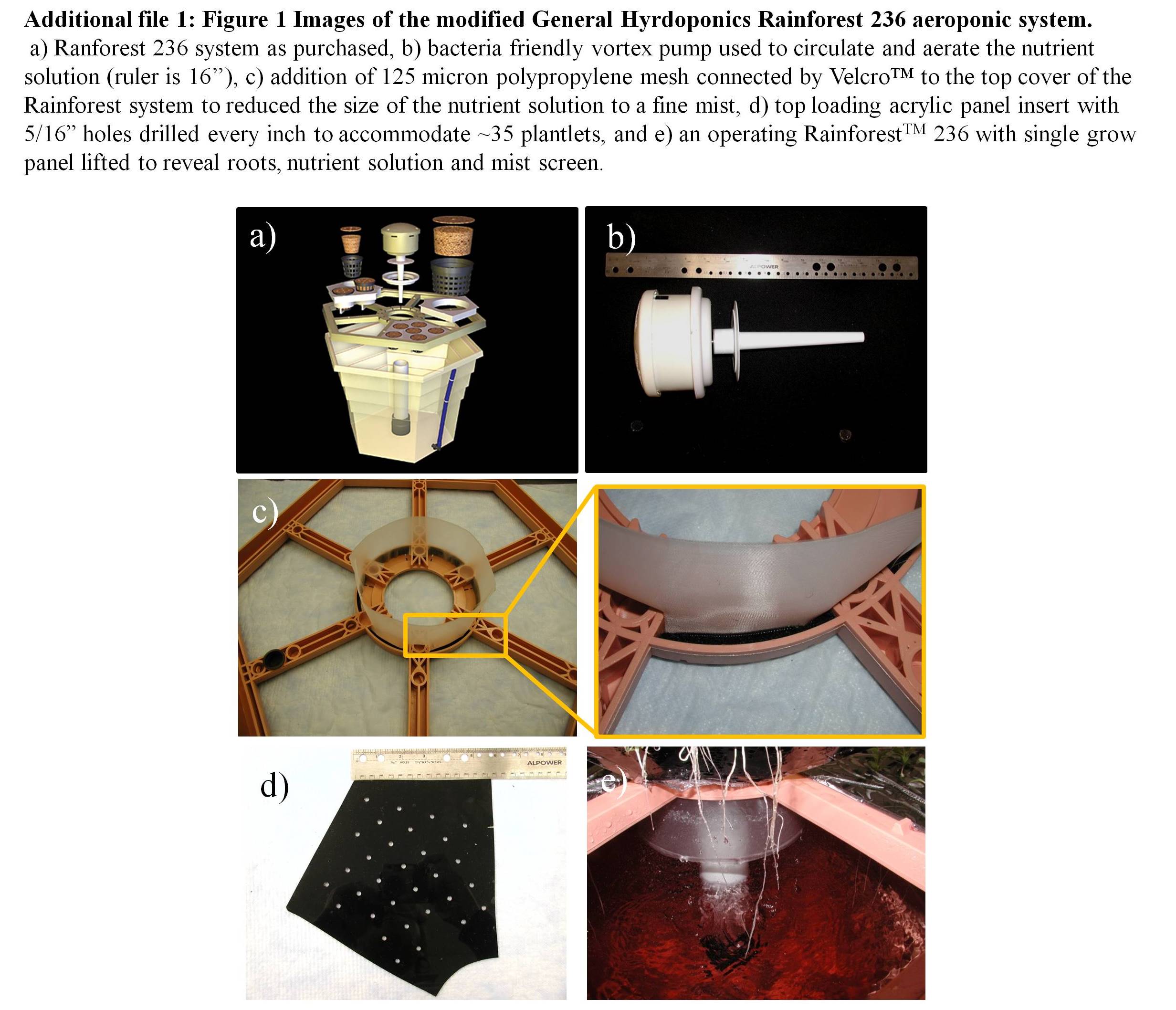

Supplement: Additional file 1 — Figure S1. Images of the modified General Hyrdoponics Rainforest 236 aeroponic system. Ranforest 236 system as purchased, b) bacteria friendly vortex pump used to circulate and aerate the nutrient solution (ruler is 16”), c) addition of 125 micron polypropylene mesh connected by Velcro™ to the top cover of the Rainforest system to reduced the size of the nutrient solution to a fine mist, d) top loading acrylic panel insert with 5/16” holes drilled every inch to accommodate ~35 plantlets, and e) an operating RainforestTM 236 with single grow panel lifted to reveal roots, nutrient solution and mist screen. [file 1756-0500-5-657-S1.jpeg]

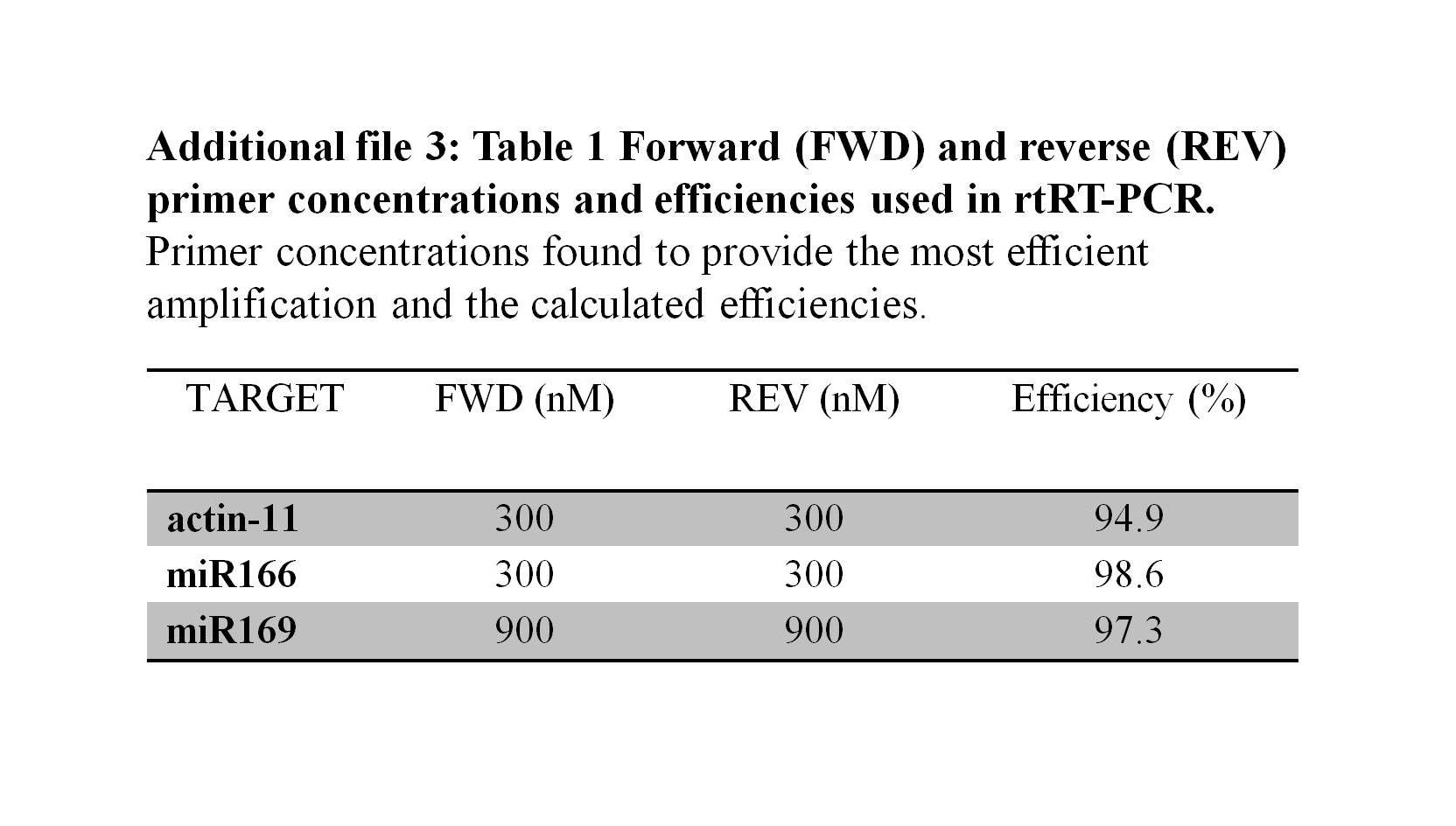

Supplement: Additional file 2 — Table S1. Forward (FWD) and reverse (REV) primer concentrations and efficiencies used in qRT-PCR. Primer concentrations found to provide the most efficient amplification and the calculated efficiencies. [file 1756-0500-5-657-S2.jpeg]

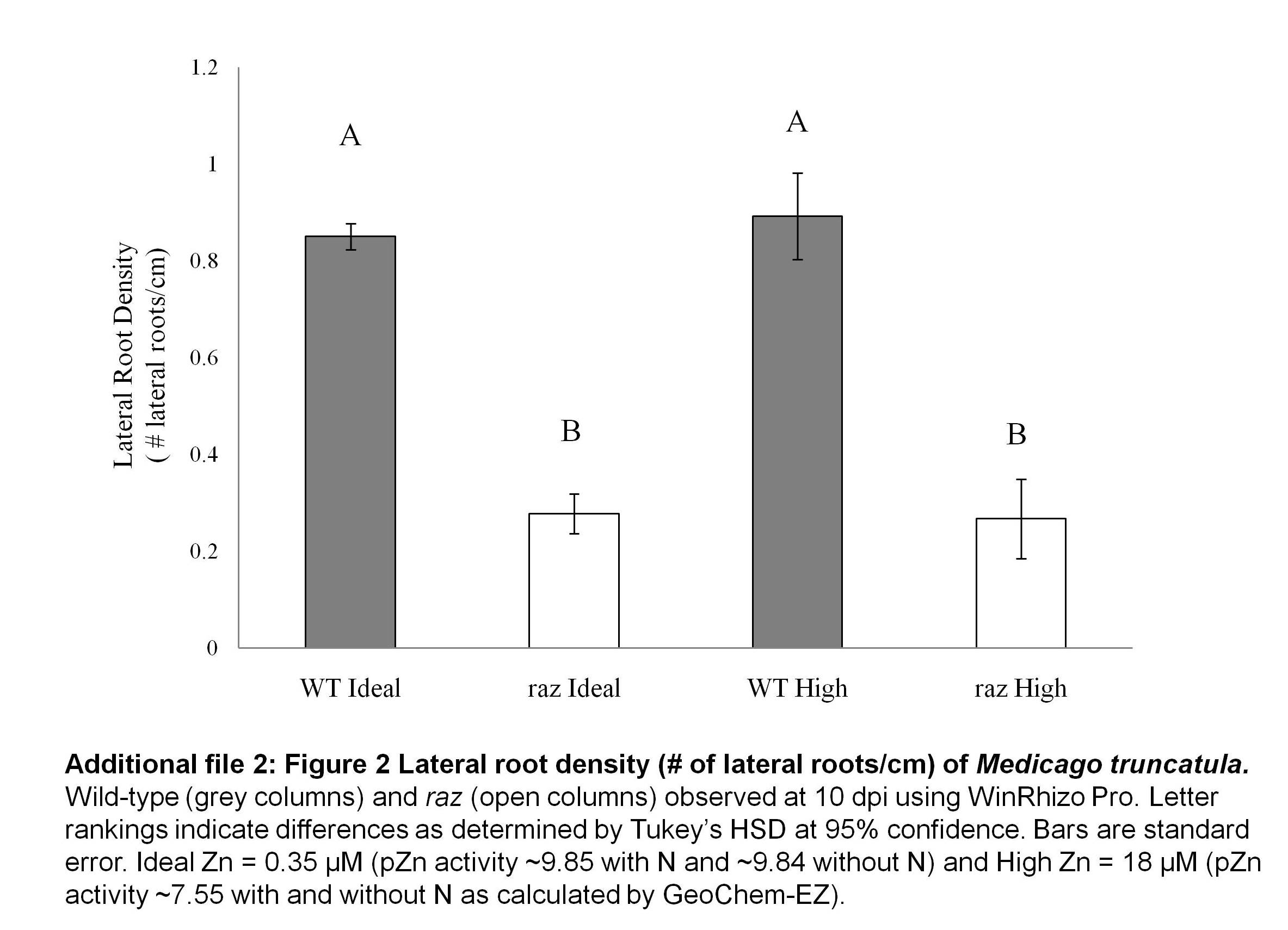

Supplement: Additional file 3 — Figure S2. Lateral root density (# of lateral roots/cm) of Medicago truncatula. Wild-type (grey columns) and raz (open columns) observed at 10 dpi using WinRhizo Pro. Letter rankings indicate differences as determined by Tukey’s HSD at 95% confidence. Bars are standard error. Ideal Zn = 0.35 μM (pZn activity ~9.85 with N and ~9.84 without N) and High Zn = 18 μM (pZn activity ~7.55 with and without N as calculated by GeoChem-EZ). [file 1756-0500-5-657-S3.jpeg]
